# Effects of Number of Animals Monitored on Representations of Cattle Group Movement Characteristics and Spatial Occupancy

**DOI:** 10.1371/journal.pone.0113117

**Published:** 2015-02-03

**Authors:** Tong Liu, Angela R. Green, Luis F. Rodríguez, Brett C. Ramirez, Daniel W. Shike

**Affiliations:** 1 Department of Agricultural and Biological Engineering, University of Illinois at Urbana-Champaign, Urbana, Illinois, United States of America; 2 Department of Animal Sciences, University of Illinois at Urbana-Champaign, Urbana, Illinois, United States of America; Institut Pluridisciplinaire Hubert Curien, FRANCE

## Abstract

The number of animals required to represent the collective characteristics of a group remains a concern in animal movement monitoring with GPS. Monitoring a subset of animals from a group instead of all animals can reduce costs and labor; however, incomplete data may cause information losses and inaccuracy in subsequent data analyses. In cattle studies, little work has been conducted to determine the number of cattle within a group needed to be instrumented considering subsequent analyses. Two different groups of cattle (a mixed group of 24 beef cows and heifers, and another group of 8 beef cows) were monitored with GPS collars at 4 min intervals on intensively managed pastures and corn residue fields in 2011. The effects of subset group size on cattle movement characterization and spatial occupancy analysis were evaluated by comparing the results between subset groups and the entire group for a variety of summarization parameters. As expected, more animals yield better results for all parameters. Results show the average group travel speed and daily travel distances are overestimated as subset group size decreases, while the average group radius is underestimated. Accuracy of group centroid locations and group radii are improved linearly as subset group size increases. A kernel density estimation was performed to quantify the spatial occupancy by cattle via GPS location data. Results show animals among the group had high similarity of spatial occupancy. Decisions regarding choosing an appropriate subset group size for monitoring depend on the specific use of data for subsequent analysis: a small subset group may be adequate for identifying areas visited by cattle; larger subset group size (e.g. subset group containing more than 75% of animals) is recommended to achieve better accuracy of group movement characteristics and spatial occupancy for the use of correlating cattle locations with other environmental factors.

## Introduction

The Global Positioning System (GPS) has become an important tool to track animal movement and behaviors. It provides researchers the capability of recording spatial and temporal information for a variety of animals [1] including mammals [2–4], reptiles [5], fish [6], and birds [7–9]. In agricultural studies, GPS technology has been utilized to study livestock behaviors and activities [10–14]. Compared to previous methods of tracking livestock locations by visual observation, GPS units worn by animals enable researchers to track animal locations at a user-specified frequency and duration, while saving labor costs, achieving higher accuracy, and yielding more complete results. With sufficient animal movement data captured by GPS, scientific models can be developed as a computational approach to study animal movement and their interactions with the environment in complex systems [15, 16]. For instance, in an integrated corn-cattle system, cattle are allowed to graze corn residues after harvest occurs. The spatial heterogeneity of field occupancy by cattle may lead to various grazing impacts on soil and subsequent crop development, which remains unclear and requires investigation [17]. Tracking cattle movement with GPS collars provides fundamental data for understanding cattle spatial occupancy and exploring corresponding impacts on agroecosystems.

Previous work regarding animal tracking with GPS has reported on GPS accuracy [18, 19], selecting appropriate GPS collar sampling frequencies [13, 20–22], and GPS errors on movement characterization and model parameterization [1, 23]. A reported and persisting challenge is labor and cost for monitoring all animals in a group [24]. There has not been sufficient research regarding the minimum number of animals required to be instrumented within a group in order to accurately preserve information representative enough for describing group behaviors [24, 25]. Further, when monitoring a subset group of animals, the extent of the information loss and its impact on subsequent analysis is unknown. Therefore, this paper will focus on the effects of subset group size on subsequent analyses in tracking cattle movements with GPS collars.

Two analytical approaches for GPS data were considered: cattle movement characterization and analysis of spatial occupancy by cattle. Movement characterization quantifies animal movement and provides information for parameterization in model development. Analysis of spatial occupancy converts GPS data into a density map that illustrates spatial patterns in grazing and helps to correlate cattle locations with their impacts on agricultural lands. This work developed a computational approach to take GPS data as inputs and quantify the impacts of the number of animals monitored on representations of cattle group movement characteristics and spatial occupancy. Our hypothesis is that there exists a subset group size that is representative of the entire herd with acceptable errors for characterization of cattle movement and cattle spatial occupancy analysis. As herd size may have influences on group movement characteristics that may yield different outcomes, this study has included two groups of cattle with different herd sizes for investigation. Results of movement data analysis were compared across subset groups and the entire herd, followed by a comparative statistical analysis to evaluate the impacts of monitoring a subset group of cattle on subsequent GPS data analysis.

## Materials and Methods

This work was conducted with approval from the University of Illinois at Urbana-Champaign Institutional Animal Care and Use Committee (IACUC, protocol #11091).

To evaluate the effects of subset group size on cattle movement analysis, this study integrated GPS technology, Geographic Information System (GIS) and other computational tools to acquire and analyze cattle location data. Grazing cattle movement data were first collected by a GPS tracking system, and then preprocessed and transformed into different data formats for further movement characterization and spatial occupancy analysis. A comparative statistical analysis was performed (OriginLab, Northampton, MA) to summarize results of the two analyses and provide suggestions for choosing an appropriate subset group size.

### 2.1 Data collection

An upgraded version of the GPS Herd Activity and Well-being Kit (GPS HAWK) tracking system for monitoring cattle locomotion [13] was implemented for this study [17]. The new design featured an updated 12-channel low-power GPS receiver (GPS18x LVC, Garmin International, Inc., Olathe, KS, USA) and generated reliable data for a five-day period at a 4 min sampling interval. The upgraded system had a lower cost per unit, which was about $283 including labor and materials. It reduced handling challenges by mounting the unit on the animal’s neck instead of the animal’s shoulder. The accuracy of the system was tested using guidelines in ION STD 101: Recommended Test Procedures for GPS Receivers [26], which showed that it had a horizontal 95% accuracy (R95) of approximately 4.0 meters. Approximate weight of a GPS unit was 1.75 kg, typically 0.3% to 0.4% of the average body weight of cattle monitored, which should not affect the behavioral patterns of the animal [12, 27].

GPS data were collected at two different study sites during the grazing seasons of 2011. The first study site was located at the University of Illinois Beef and Sheep Field Research Laboratory (lat 40^◦^ 04.7’ N, long 88^◦^ 13.8’ W; elevation 215m), a research farm (owned and managed by University of Illinois) contiguous to the Urbana-Champaign campus. An 80 hectare pasture was dedicated to rotational grazing of beef cattle, with approximately 1.53 to 1.83 hectares for each paddock. A mixed group of 24 beef cows and heifers (group A, mean body weight 452.2 24.9(SD) kg) were monitored on fenced fescue pastures by GPS collars for seven consecutive weeks during the summer grazing season of 2011. Water was provided using a small tank located at the southwest corner of the paddock with a maximum walking distance of 380 m. The second study site was located at the Dudley Smith Farm (lat 39^◦^ 26.4’ N, long 89^◦^ 07.1’ W; elevation 202m), 6 km northwest of Pana, Illinois. The Dudley Smith Farm is owned by the University of Illinois, and jointly managed by the Office of Research, within the College of Agriculture Consumer and Environmental Sciences, and the Dudley Smith Initiative. Cattle were allowed to graze corn crop residues on the cropland after harvest. Strip grazing management was conducted for 42 days, such that the field was divided into three strips using electric fences and cattle were allowed access to the next strip every 14 days. A separate analysis was conducted to determine whether strip grazing management could preserve forage quality as compared to continuous grazing management that allows cattle to access the entire field during grazing. The second group (group B, mean body weight 526.4 6.7(SD) kg) including 8 Angus heifers was monitored on the corn residue fields for six consecutive weeks during the fall grazing season of 2011. Water was provided using a small tank located at the northwest corner of the first strip with a maximum walking distance of 200 m. Based on the data availability and quality, two sets of GPS data were selected from the two groups respectively: GPS records beginning at 00:00 June 2, 2011 and ending at 00:00 June 10, 2011 (eight consecutive days) were selected for analysis of group A; GPS records beginning from 10:00 October 24, 2011 and ending at 00:00 October 28, 2011 (approximately three and a half consecutive days) were selected for analysis of group B. For both selected periods, cattle were grazing within the same paddocks at their respective farm locations. Approximately 1.83 hectares of pasture (a 96 m × 378 m × 390 m right triangular paddock) was available to group A, and approximately 1.75 hectares corn residue land (a 68 m × 257 m rectangular paddock) was available to group B during their respective periods. Both studies were conducted on flat areas under no canopy (variations of elevation < 3 m). During the course of the studies, GPS units were never dislodged by the animals. GPS accuracy was tested and validated before each deployment. Damaged or inaccurate GPS units were replaced and removed from use.

For both groups, an initial adaptation period was implemented to allow cattle to adjust to wearing weighted collars before fitting GPS instrumentation. Approximately one week before GPS data were collected, cattle were sequentially fitted with a collar and empty equipment box for 1 to 2 days; then a collar and weighted equipment box (similar weight to the GPS collar) for 2 to 4 days; then with functioning GPS for data collection. Collars were secured such that they did not hang loosely or swing from below the neck, but still allowed at least one hand easily placed between the collar strap and the side of the neck. With each subsequent collar placement, fit was adjusted (if needed) to minimize slack in the strap, while avoiding any buildup of fluid beneath the neck. In previous tests, this adaptation period and fit was shown to be effective to reduce shaking and rubbing that resulted in damage to GPS equipment or problems for the animals.

GPS data were collected at a specified sampling interval of four minutes. To reduce the risk of data loss, data were collected and stored as space delimited text (.txt) files every hour. Each GPS location fix (date, time, latitude, longitude, number of satellites in view, and differential correction status) was held in temporary memory and written to removable compact flash storage after 15 consecutive GPS location fixes were collected; a new file was created for every save event. Spatial coordinates were calculated using the World Geodetic System 1984 earth datum, and the receiver output the date and time in Coordinated Universal Time.

### 2.2 Data preprocessing

All GPS data were downloaded from GPS collars after each collection period. A C++ program was used to merge all the text files into one comma-separated-value (.csv) file while preserving GPS information for each animal. In addition, the program removed corrupted text, and converted date and time from Coordinated Universal Time to Central Standard Time, the time zone of the two study sites. The csv files were then imported to ArcGIS (ESRI, Redlands, CA) and stored as shapefiles for visualization and analysis. Location data were converted from latitude and longitude to the Universal Transverse Mercator (NAD UTM 1983 zone 16N) format to enable calculations of algebraic derivation of distance between locations. Aerial maps were downloaded as basemaps from ArcGIS online database to be overlaid with cattle movement data for further analysis. Meanwhile, cattle location data stored as UTM format were exported as dBASE Table files for further computation and analysis in MATLAB (R2010b, The MathWorks, Natick, MA).

### 2.3 Cattle movement characterization

**2.3.1 Data synchronization**. As GPS data were not recorded at exactly the same time throughout the experiment, in order to calculate herd movement parameters, a linear interpolation algorithm was used to align the data to the same timeline using the sampling interval. To manage data loss during the experiments, if a GPS receiver failed to receive location data for more than twenty minutes (five consecutive potential records), that portion of the dataset was not interpolated and the animal with that GPS receiver was removed from calculations of group movement parameters for that period of time. A matrix was used to store the number of GPS receivers that had records at each time stamp. For group A, the average number of location fixes collected per day per animal was 318 (about 87.0% of expected location fixes). Group B acquired almost all data of expected fixes (only 9 fixes lost for the entire group during the whole study period), as each animal had about 363 fixes per day.

**2.3.2 Generating subset groups**. Subset groups were generated by randomly using different combinations of animals. For group A, subset group sizes were generated ranging from 4 to 20 animals with an increment of one. Given a subset group size k, the subset group should contain k distinct cows from group A, and the number of k-combinations is equal to the binomial coefficient C(24, k). Since group A had 24 animals in total, even the smallest number of k-combinations (C(24,4) or C(24,20)) is 10,626, while the largest number (C(24,12)) is 2,704,156. Therefore, instead of considering all possible combinations, 1000 unique random subset groups, a number large enough to represent the characteristics of the population, were generated for each subset group size of group A. Similarly, for group B, subset group sizes were generated ranging from 1 to 7 with the increment of one. All possible subset groups for each subset group size were considered (largest number of k-combinations is 70).

**2.3.3 Calculating movement metrics**. Based on previously published papers related to characterization or modeling of ungulate movement [4, 12, 13, 28–31], a set of individual and group movement parameters were defined to characterize cattle movement from GPS data for this analysis, including individual cow travel speed V_n, i_ (m/min), herd centroid coordinates (${\text{X}}_{\text{n}}^{\text{c}}$,${\text{Y}}_{\text{n}}^{\text{c}}$), herd travel speed${\text{V}}_{\text{n}}^{\text{c}}$ (m/min), cow distance to centroid D_n, i_ (m), herd radius ${\text{R}}_{\text{n}}^{\text{c}}$(m), and herd cumulative travel distance ${\text{L}}_{\text{n}}^{\text{c}}$(m), where n denotes the n^th^ time stamp, ranging from 1 to N, and i denotes the cow ID.

Individual cow travel speed V_n, i_ was defined as the Euclidean distance between two consecutive GPS locations of an animal divided by the GPS sampling interval Δt (Eq. 1). It does not represent the actual animal movement speed, but quantifies the measured displacement of animal locations over time.

$${\text{V}}_{\text{n,i}}=\frac{\sqrt{{\left({\text{X}}_{\text{n,i}}-{\text{X}}_{\text{n-1,i}}\right)}^{2}+{\left({\text{Y}}_{\text{n,i}}-{\text{Y}}_{\text{n-1,i}}\right)}^{2}}}{\text{Δt}}$$ (1)

X_n, i_, Y_n, i_—UTM coordinates of cow i at the n^th^ time stamp

Herd centroid coordinate ($X_{n}^{c}$,$Y_{n}^{c}$) was defined as the geometric center of the herd; and was calculated by averaging the coordinates of all animals with GPS records at the n^th^ time stamp (Eq. 2).

$${\text{X}}_{\text{n}}^{\text{c}}=\frac{\underset{\text{i}}{\sum }{\text{X}}_{\text{n,i}}}{{\text{I}}_{\text{n}}},{\text{Y}}_{\text{n}}^{\text{c}}=\frac{\underset{\text{i}}{\sum }{\text{Y}}_{\text{n,i}}}{{\text{I}}_{\text{n}}}$$ (2)

I_n_–Number of animals with GPS records at the n^th^ time stamp

Similar to individual animal travel speed, herd centroid speed $V_{n}^{c}$ was defined as the Euclidean distance between two consecutive locations of the herd centroid divided by the sampling interval ∆t (Eq. 3).

$${\text{V}}_{\text{n}}^{\text{c}}=\frac{\sqrt{{\left({\text{X}}_{\text{n}}^{\text{c}}-{\text{X}}_{\text{n-1}}^{\text{c}}\right)}^{2}+{\left({\text{Y}}_{\text{n}}^{\text{c}}-{\text{Y}}_{\text{n-1}}^{\text{c}}\right)}^{2}}}{\text{Δt}}$$ (3)

Animal distance to centroid $D_{n,i}$ was defined as the Euclidean distance between cow i and the herd centroid at the n^th^ time stamp (Eq. 4).

$${\text{D}}_{\text{n,i}}=\sqrt{{\left({\text{X}}_{\text{n,i}}-{\text{X}}_{\text{n}}^{\text{c}}\right)}^{2}+{\left({\text{Y}}_{\text{n,i}}-{\text{Y}}_{\text{n}}^{\text{c}}\right)}^{2}}$$ (4)

Further, herd radius $R_{n}^{c}$ was defined as the average distance to herd centroid for all cattle in the group at the n^th^ time stamp (Eq. 5).

$$R_{n}^{c}=\frac{\sum_{i}D_{n.i}}{I_{n}}$$ (5)

Herd cumulative travel distance $L_{n}^{c}$ was defined as the cumulative distance traveled by the herd centroid at the n^th^ time stamp (Eq. 6).

$${\text{L}}_{\text{n}}^{\text{c}}=\overset{\text{t=n}}{\underset{\text{t=2}}{\sum }}\sqrt{{\left({\text{X}}_{\text{t}}^{\text{c}}-{\text{X}}_{\text{t-1}}^{\text{c}}\right)}^{2}+{\left({\text{Y}}_{\text{t}}^{\text{c}}-{\text{Y}}_{\text{t-1}}^{\text{c}}\right)}^{2}}$$ (6)

All the parameters defined above were stored as time series that were calculated at every time stamp. Mean values over the whole period were calculated including average herd travel speed ${\overset{-}{\text{V}}}^{c}$(m/min), average herd radius ${\overset{-}{\text{R}}}^{c}$(m), and average herd daily travel distance ${\overset{-}{\text{L}}}^{c}$(m/day) for comparison between subset groups and the entire group. Additionally, average centroid location deviation ${\overset{-}{\text{C}}}_{\text{dev}}$(m) and average herd radius deviation ${\overset{-}{\text{R}}}_{\text{dev}}$(m) were calculated to quantify the average errors caused by subsampling.

Average herd travel speed ${\overset{-}{\text{V}}}^{c}$ was defined as the mean value of centroid speed over the whole period (Eq. 7).

$${\bar{V}}^{c}=\frac{\overset{n=N}{\underset{n=1}{\sum }}V_{n}^{c}}{N}$$ (7)

N–Total number of time stamps

Average herd daily travel distance ${\overset{-}{\text{L}}}^{c}$ was defined as the mean daily distance traveled by the centroid of herd over the whole period (Eq. 8).

$${\bar{L}}^{c}=\frac{L_{N}^{c}}{T_{N}}$$ (8)

$T_{N}$- Total period length (day)

Average herd radius ${\overset{-}{\text{R}}}^{c}$ was defined as the mean herd radius of the group over the whole period (Eq. 9).

$${\bar{R}}^{c}=\frac{\sum_{n=1}^{n=N}R_{n}^{c}}{N}$$ (9)

Average centroid location deviation ${\overset{-}{\text{C}}}_{\text{dev}}$ was defined as the mean distance between the centroids of subset group and the entire herd over the whole period (Eq. 10).

$${\bar{C}}_{dev}=\frac{\sum_{n=1}^{n=N}\sqrt{{\left(X_{n}^{c\prime }-X_{n}^{c}\right)}^{2}+{\left(Y_{n}^{c\prime }-Y_{n}^{c}\right)}^{2}}}{N}$$ (10)

($X_{n}^{c\prime }$,$Y_{n}^{c\prime }$)—Centroid coordinates of the subset group at the n^th^ time stamp

Average herd radius deviation ${\overset{-}{\text{R}}}_{\text{dev}}$ was defined as the mean deviation between subset group and herd radii over the whole period (Eq. 11).

$${\bar{R}}_{dev}=\frac{\sum_{n=1}^{n=N}\sqrt{{\left(R_{n}^{c\prime }-R_{n}^{c}\right)}^{2}}}{N}$$ (11)

$R_{n}^{c\prime }$- Herd radius of the subset group at the n^th^ time stamp.

### 2.4 Spatial occupancy analysis

**2.4.1 Generating subset groups**. Because group A had a larger herd size than group B, group A was considered a better demonstration of the results for a wider range of subset group size (similar results have been observed for group B). As described in 2.3.2, the number of possible combinations of subgroups of group A is very large. Due to limitations (e.g. data storages and processing time), a selection of possible combinations was included in this analysis, consisting of subset group sizes ranging from 4 to 20 with an increment of 2. For each subset group size, five different random subset groups were selected for analysis.

**2.4.2 Kernel density estimation**. A kernel density estimation (KDE) was performed to convert GPS point data to a raster map that quantifies cattle visitation rates at specific locations throughout the fields. This allows us to consider herd impacts upon field characteristics. For each cattle location recorded by GPS collars, a smooth curved surface based on the kernel function was fitted [32], with the highest surface value at the location of the point, with diminishing values as distance increases from the point, and with zero value at the limit of the search radius. The density at each output raster cell was calculated by adding the values of all the kernel surfaces where they overlay the raster cell center. The GPS R95 accuracy (4 m) was used as the search radius, with output raster map cell size of 2 m by 2 m.

**Fig 1:**
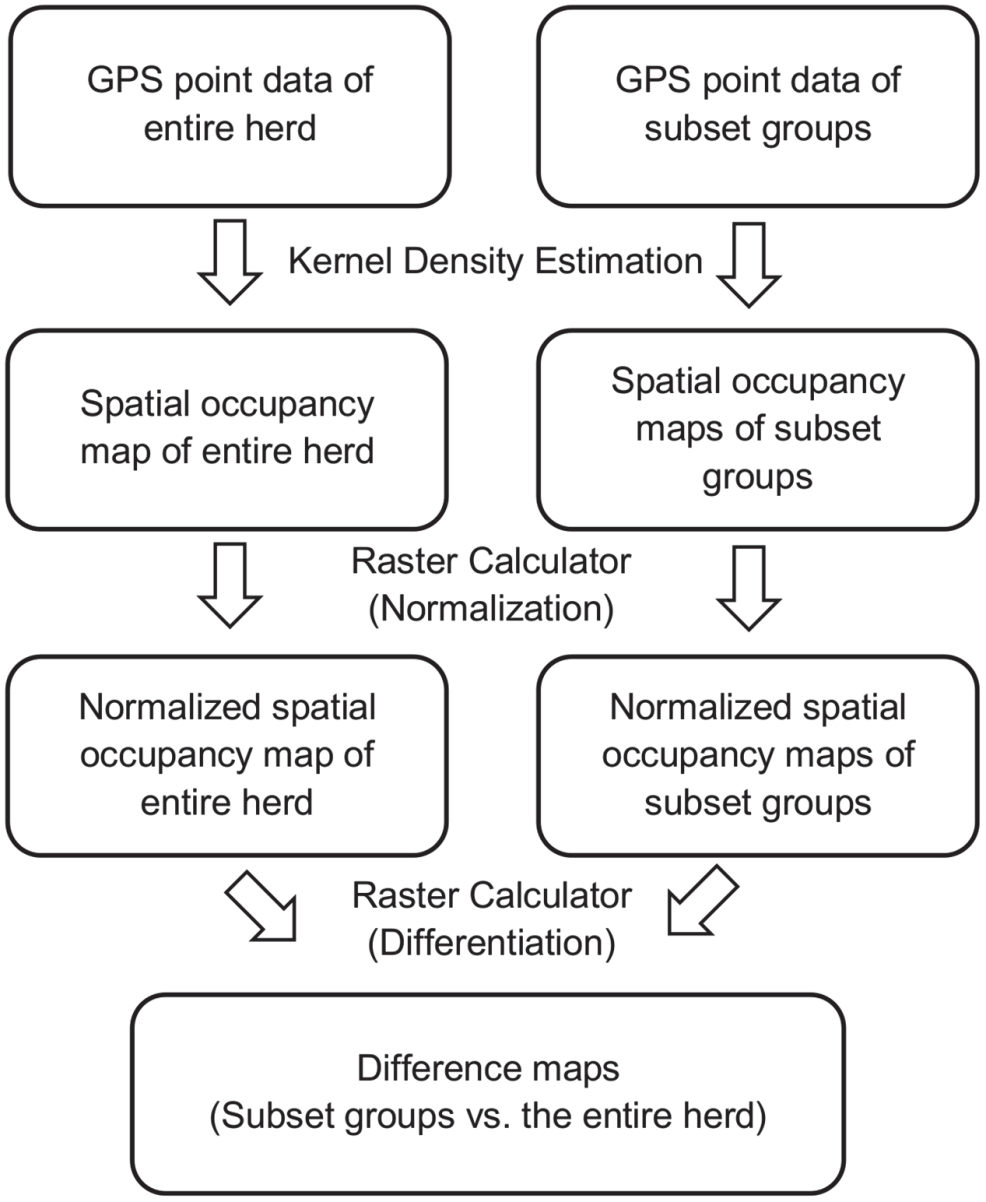
Geoprocessing workflow for comparing KDE maps of subset groups with the entire herd. ArcGIS ModelBuilder (ESRI, Redlands, CA) was used to facilitate the spatial comparison of density maps of grazing distributions between subset groups and the entire herd via GPS point data.

The geoprocessing workflow in ArcGIS includes generation and comparison of normalized density maps between subset groups and the entire herd (Fig. 1). Kernel density estimation was performed for subset groups and the entire herd. Then, the density maps were clipped to ensure that all maps had the same domain. Since the range of output raster cell values was different for different subset groups, for each density map, all cell values were normalized linearly to a common range (0 to 1) for comparison. Finally, these normalized density maps were compared between subset groups and the entire herd based on their spatial locations, and new raster maps were created that denoted the differences. The cell values in the new maps were equal to the differences between subset groups and the entire herd.

### 2.5 Comparative statistical analysis

For each subset group size, there were many different combinations of animals to form the subset group; thus, an error tolerance was introduced to evaluate the effects of subset group size on the results of movement characterization and spatial occupancy analysis statistically based on whether the subset group’s results lie within the error tolerance.

For average herd travel speed, average herd daily travel distance, and average herd radius, the entire herd’s results were assigned as the “true” value, and the error tolerance was defined as an interval with a ±5% bandwidth of the true value. For average centroid location deviation and average herd radius deviation, the GPS R95 accuracy (4.0 m) was used as the error tolerance. Any results that were below R95 were considered acceptable because the source of this error cannot be distinguished between the limitation of GPS accuracy or by using the subset group instead of the entire herd. For each subset group size, the percentages (S) of results calculated by subset groups that were within the error tolerance were calculated (Eq. 12 and Eq. 13).

$$S=\frac{N_{\left|errors\leq 5\%\right|}}{N_{total}}\times 100\%$$ (12)

$$\text{S}=\frac{{\text{N}}_{\text{errors}\leq \text{R}95}}{{\text{N}}_{\text{total}}}\times 100\%$$ (13)

In spatial occupancy analysis, a normalized KDE map illustrated the field visitations by cattle. Further, a difference map was created to denote the differences between subset group and the entire herd. The cell values of this map were close to zero if KDE maps of subset groups and the entire herd were similar. Likewise, an error tolerance of ±0.05 was introduced, and the percentage (S) of cells in the difference map whose value is less than or equal to 0.05 was calculated (Eq. 14).

$$\text{S}=\frac{{\text{N}}_{\left|\text{errors}\right|\leq 0.05}}{{\text{N}}_{\text{total}}}\times 100\%$$ (14)

S–The percentage of instances (subset groups in movement characterization) or cells (KDE maps in spatial occupancy analysis) within the error tolerance.

N_total_–Total number of instances (subset groups in movement characterization) or cells (KDE maps in spatial occupancy analysis).

N_|errors|≤5%_–Number of instances within the error tolerance that is an interval with a ±5% bandwidth off the true value.

N_errors≤R95_—Number of instances within the error tolerance that is below the GPS R95 accuracy

N_|errors|≤0.05_—Number of cells within the error tolerance in the KDE map.

## Results and Discussion

### 3.1 Effects of subset group sizes on movement characterization

**Fig 2:**
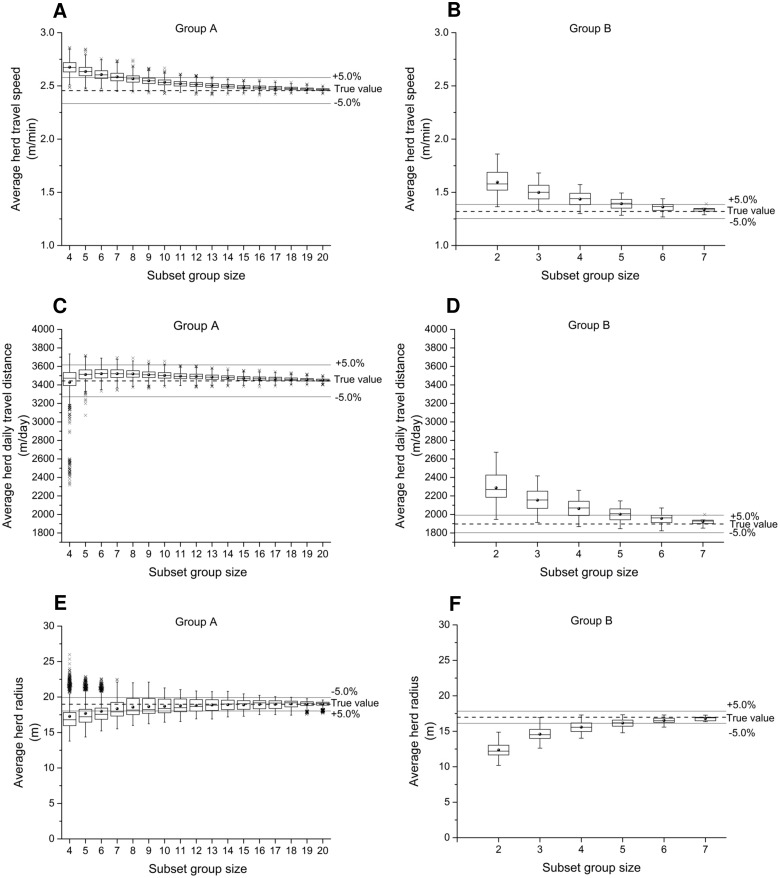
Effects of subset group size on measurement of group movement parameters. Average herd travel speed (A and B), daily travel distance (C and D) and herd radius (E and F) of subset groups are compared with the entire herd (denoted as “true value” in the figures). First, second and third quartiles are displayed by boxes, the range by whiskers, mean values by solid dots, and outlying values by crosses. A bandwidth of ±5% of the true value is used as the error tolerance.

**3.1.1 Average herd travel speed**. Cattle movement can be classified into three movement modes: resting, traveling, and foraging [12, 15, 31], which results in different patterns in group movement and individual movement. During resting, both individual animal speeds and herd centroid speed are close to zero; when the herd is traveling from one location to another location (e.g. a water station), animals move approximately at the same speed, therefore the herd speed is close to individual speeds; while animals are foraging, herd travel speed may be quite different from, usually lower than, individual animals speeds. As subset group size decreases, herd centroid movement tends toward individual animal movements and selection of different subset groups generates a wider range of variability within results due to variations of animals within the herd (Fig. 2A and 2B). As the subset group sizes decreases, for both group A and B, the mean values of average herd centroid travel speed increase, as well as the standard deviations.

**3.1.2 Average herd daily travel distance**. Travel distances are associated with travel speeds; hence, similar to average herd travel speed, means and standard deviations of herd daily travel distance also increase as the subset group size decreases. A notable difference between group A and B is that group A has an increasing number of outliers (denoted as crosses) when subset group size decreases as compared to group B (Fig. 2C and 2D). The magnitude of most outliers were smaller than the “true” value of average herd daily travel distance, calculated using the entire herd, which is in conflict with the trend shown in Fig. 2C and 2D that decreasing subset group size leads to potential overestimation of average herd daily travel distance. This was caused by GPS data loss during monitoring. It is very common in animal monitoring studies with GPS collars that GPS units undergo intermittent failure due to many reasons such as bad weather, poor satellite signal, damage caused by animals, and so on. As group A suffered more GPS data loss during data collection than group B (about 87% of expected fixes were collected for group A while nearly all expected fixes were collected for group B), the data loss becomes increasingly impactful in estimation of travel distance when subset group size is small. Impacts of GPS data loss during collection can be considered similar to increasing data sampling frequency for that period of loss, as fewer or no location fixes are recorded. Several studies [13, 20–22] have shown that animal daily travel distance estimated from GPS data can significantly decrease when GPS sampling frequency was decreased. This helps explain why loss of GPS data leads to the underestimation of average herd daily travel distances. Therefore, monitoring a small subset group of animals has a greater probability to cause significant inaccuracy in estimation of herd daily travel distances, which is further increased if data loss occurs during collection.

**3.1.3 Average herd radius**. As the subset group size decreases, the mean of average herd radius over time is less than the true value while the standard deviation increases (Fig. 2E and 2F). Herd radius is calculated by averaging individual distance to the herd centroid (an alternative movement parameter is the average distance between individual animals [12]). Thus, it can be considered as a magnitude that denotes the range of the herd, which is often associated with the areas visited by cattle. In addition, herd radius may vary for different cattle activities. For instance, cattle usually have a larger radius when cows are spread out over pastures for grazing or during traveling as compared to resting [12]. Therefore, average herd radius can be used as a factor for distinguishing different cattle behaviors based on GPS data. In modeling, herd radius can also be used as a parameter that specifies the range of the herd during different activities to simulate synchronized group behaviors (e.g. traveling, foraging and resting). The results suggest that using a subset group for monitoring could potentially underestimate herd radius, thus this may introduce errors when classifying cattle behaviors.

**Fig 3:**
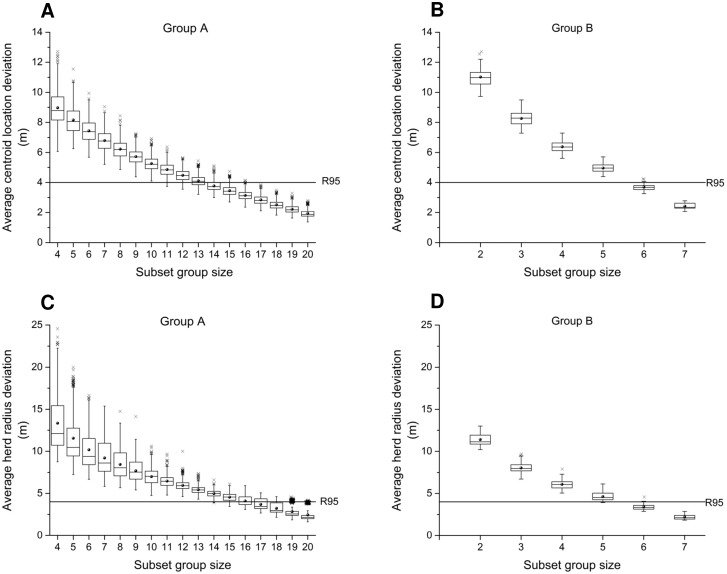
Effects of subset group size on deviation of herd centroid location and herd radius. Average centroid location deviations (A and B) and herd radius deviations (C and D) are compared with GPS accuracy (R95). First, second and third quartiles are displayed by boxes, the range by whiskers, mean values by solid dots, and outlying values by crosses. GPS R95 accuracy is used as the error tolerance.

**3.1.4 Average centroid location deviation and average herd radius deviation**. Both average centroid location deviation and average herd radius deviation quantify the differences between subset groups and the entire herd at every time step. Average centroid location deviation gives the distances between the two centroids of subset groups and the whole herd. Average herd radius deviations denote the differences of herd radius between a subset herd and the entire herd. Both groups’ mean deviation linearly increases ($r_{A}^{2}=0.973$ and $r_{B}^{2}=0.973$) as subset group size decreases. As subset size decreases, the individual variability has more impacts on subsampling, which leads to an increase of standard deviation. Using GPS R95 as the error tolerance (horizontal lines in Fig. 3) requires a subset group containing at least 16 animals to have all the deviations within R95 for group A (Fig. 3A). Similarly, at least 6 animals are required for a group of eight animals, as in group B (Fig. 3B). Average herd radius deviation and its standard deviation also increase linearly ($r_{A}^{2}=0.950$ and $r_{B}^{2}=0.953$) as subset group size decreases. Using GPS R95 accuracy as the error tolerance shows that subset groups including at least 19 cows from group A and 6 cows from group B have nearly all errors within R95 (Fig. 3C and 3D).

**Fig 4:**
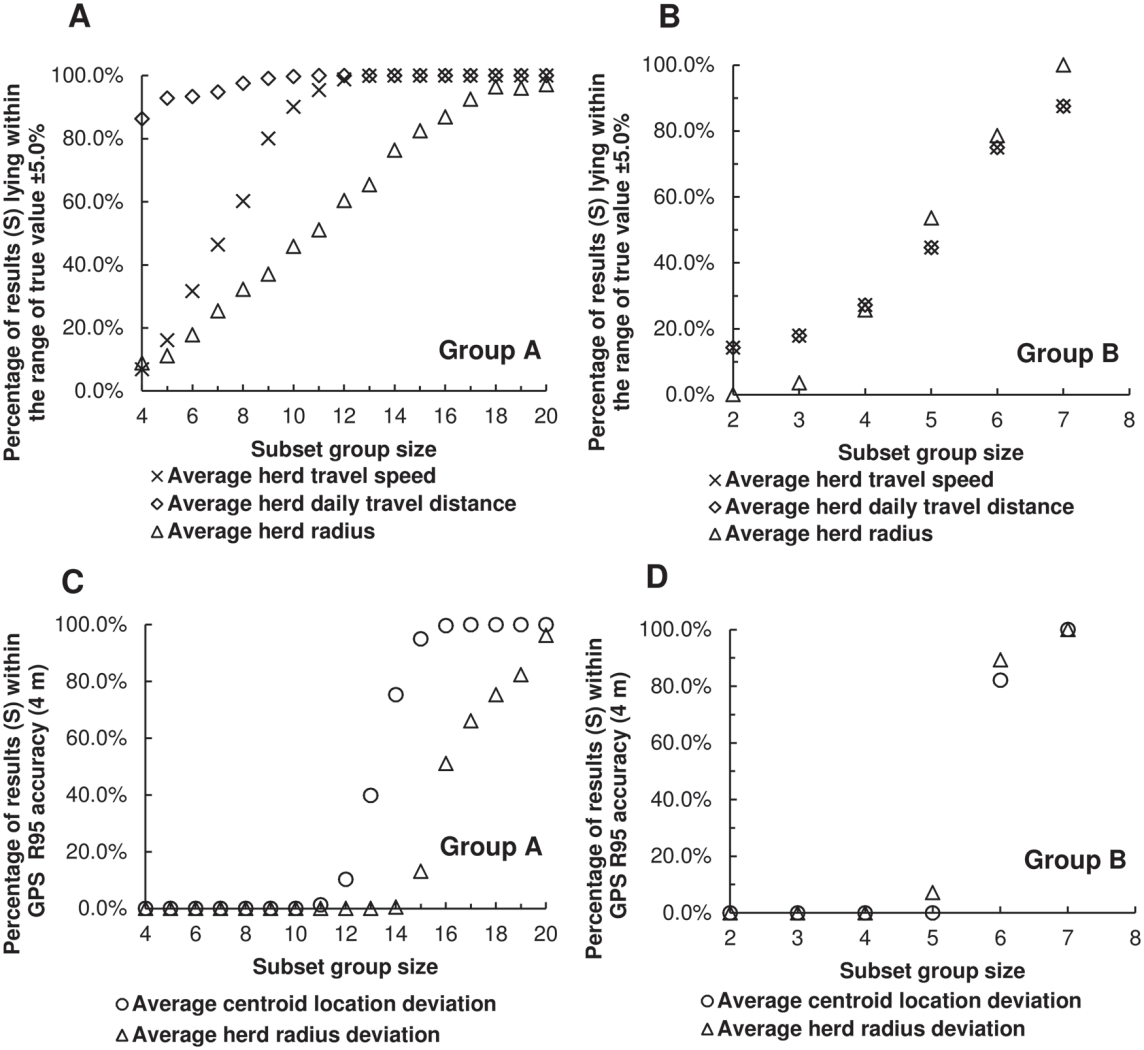
Comparative statistics for error evaluation of movement characterization. The results illustrate the percentage of subset groups that are within the error tolerances: percentage of results within the range of the true value ±5% for average herd travel speed, average herd daily travel distance, and average herd radius (A and B); percentage of results within R95 for average centroid location deviation and average herd radius deviation (C and D).

**3.1.4 Comparative statistics**. For both group A and B, S increases as subset group size increases, approaching 100% as subset size gets close to the herd size (Fig. 4). Different patterns of average herd travel speed and daily travel distance were found between group A and B (Fig. 4A and 4B). For group A, which had a relatively large herd size, and worse GPS data quality compared to group B, the increase of S can be distinguished into two phases as subset group size increases. When group size is small, S increases rapidly during the first phase, and then slightly increases during the second phase. For a subset group size that is greater than 12, about 95% of subset groups’ average herd travel speed and daily travel distance are within the error tolerance range as compared to the entire herd. When considering average herd radius and a small subset group size, a lower percentage of subset groups within error tolerance were reported for group A (Fig. 4A). To achieve a greater percentage of instances within the error tolerance (i.e. 95%), monitoring 18 cows from group A is required. For group B, which had a nearly complete dataset of location fixes, S values for the average herd travel speed and daily travel distance were linearly correlated (crosses and diamonds in Fig. 4B), because travel distance can be calculated using travel speed and time interval if no data loss occurs. Unlike group A, S values for the three movement parameters of group B increase steadily as subset group size increases (Fig. 4B). These different patterns between group A and B (Fig. 4A and 4B) may result from several factors including treatment differences that could affect animal movement (e.g. different forage types, grazing seasons, water demand, weather and shape of paddocks), different herd sizes, and varying levels of completeness of GPS data collected. However, results of group A and B both indicate that selecting 75% animals of the entire herd will maintain errors within tolerance for all three movement parameters with 80% probability.

Similar patterns of S were found for average centroid location deviation and average herd radius deviation (Fig. 4C and 4D). One difference between group A and B is that, for group A, the increase of S for average centroid location deviation decreases after the subset group size is large enough (e.g. 16) and S is greater than 90%. This is similar to the patterns observed with average herd travel speed and average herd daily travel distance (Fig. 4A), which indicate that increasing subset group size, when subset group size is large enough (e.g. at least 16 cows), will not make significant improvement in the results as errors become very small.

Appropriate animal subset group size to monitor for modeling purposes depends on many factors, such as the movement characteristics one would like to obtain, as well as tolerance for error. In summary, results of two groups suggest that there is a greater inaccuracy in movement characterizations for monitoring a subset group, when the entire herd size is small. For instance, monitoring 7 animals out of 8 has about 15% chance to cause a relative error of more than 5% of the true value for herd travel speed and daily travel distance. However, for cattle groups with greater sizes, there may be a proper subset group size that can generate results close to the true values. For instance, monitoring 20 out of 24 cows has about 95% chance to maintain the errors of movement parameters within 5% of the true values.

### 3.2 Effects of subset group sizes on spatial occupancy analysis

**Fig 5:**
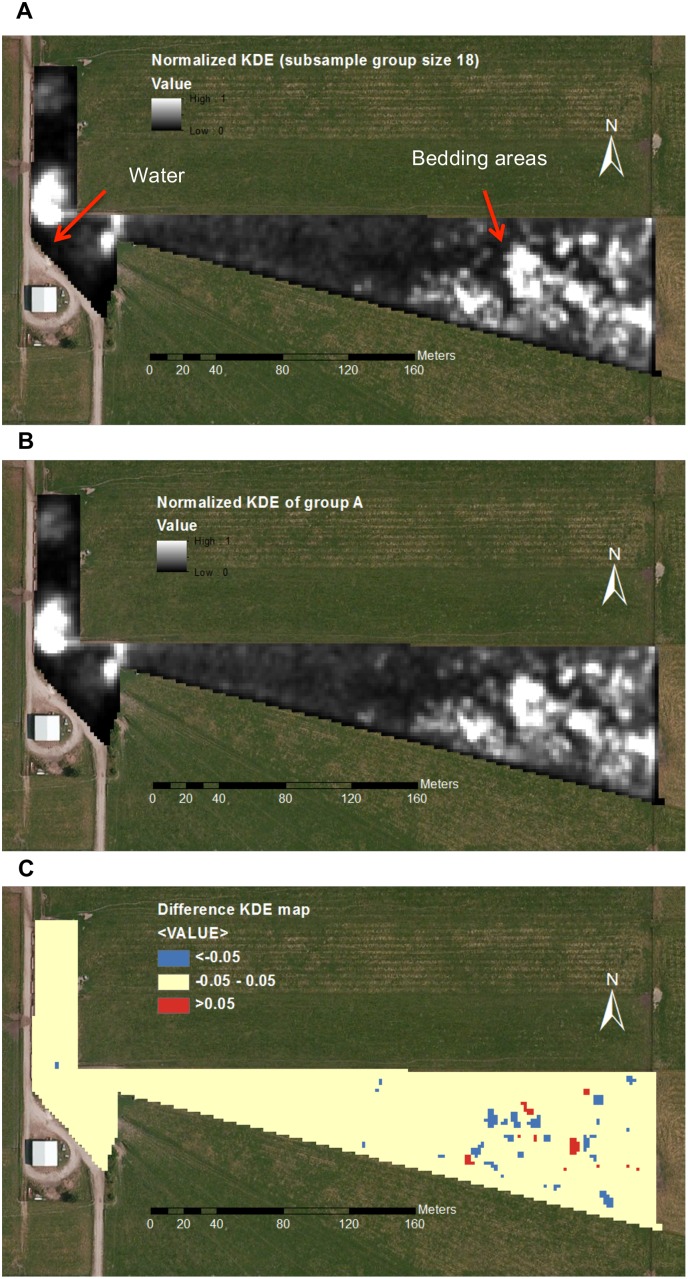
Comparison of KDE maps between subset groups and the entire herd. Example of GPS location data of a subset group of size 18 (group A) were converted to a rasterized map, which quantifies the pasture visitation rates by cattle using KDE analysis. White and black areas denote the grazing areas for the herd. Intense white areas are indicative of high cattle visitation rates. The map was further normalized (A) in order to compare with a similar map (B) of the entire herd. A difference map (C) was created to denote the differences between the two maps (pixels with values larger than 0.05 are shown in red, smaller than -0.05 in blue, and others in beige). The same highly visited areas can be identified based on (A) or (B) since they are very similar spatially, as white intense areas on both maps are located around watering and bedding areas.

Cattle are usually regarded as social animals as they prefer to live together in groups, and members can be highly interactive with each other. Their behaviors are often coordinated in time (synchronization) and in space (cohesion of the herd) [12], and this leads to the phenomenon that they usually have the same favored resting areas, feeding sites and water locations [15, 20]. In the spatial occupancy analysis, results show that instead of randomly visiting areas within the pasture, animals repeatedly visited certain areas, which leads to a spatially uneven location visitation by cattle (Fig. 5). Previous studies have shown that grazing animals living in social groups often have synchronized activities and behaviors [12, 33–35], which can lead to similar distributions of spatial occupancy of land among individuals. The KDE results of the spatial occupancy analysis show that individual animals, subset groups, and the entire herd have similar spatial patterns (Fig. 5). The highly visited areas (intense white area in Fig. 5) identified from the KDE maps can be classified as either bedding areas, feeders, water stations or foraging areas.

**Fig 6:**
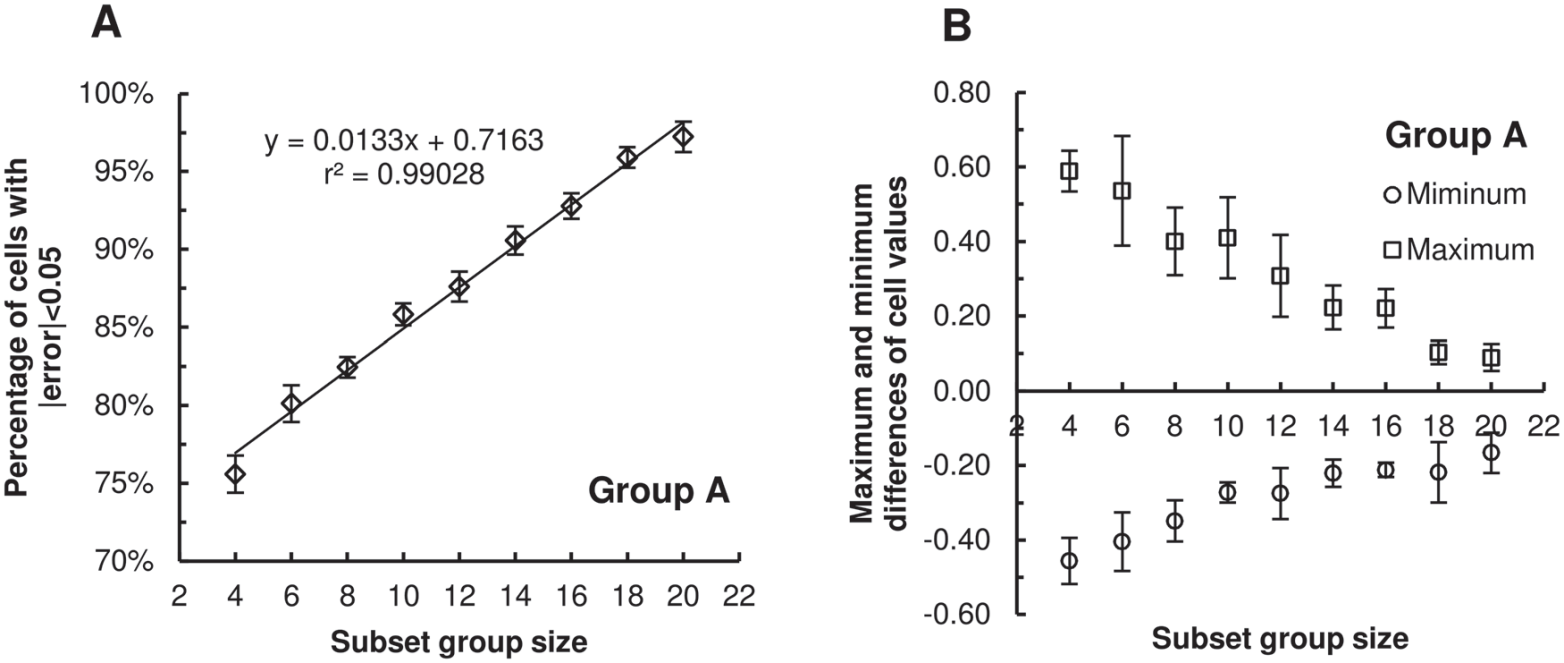
Comparative statistics for error evaluation of spatial occupancy analysis. The average percentage of cells within difference maps within the error tolerance (|errors|<0.05) increases linearly (*r* ^*2*^ = 0.99028) as subset group size increases (A). The average maximum and minimum difference values decreases as subset group size increases (B). Error bars denote ± SE.

As described in section 2.5, the accuracy of cattle spatial occupancy analysis was evaluated using a subset group by creating a difference map to denote the differences between the subset group’s field visitation map and the entire herd. The similarity of two KDE maps was investigated by counting the percentage (denoted as S in Eq. 14) of cells with values that are within the error tolerance (0.05) in the difference map. This analysis suggests that S linearly increases as the subset group size increases (Fig. 6A). For instance, S reaches 95% when the subset group size is 18; thus, the spatial occupancy map of a subset group of size 18 is about 95% similar to that of the entire herd of size 24 given an error tolerance of 5%. Further, the absolute values of both maximum and minimum cell values in the difference maps decrease as the subset group size increases (Fig. 6B). Results of spatial occupancy rates also show that using a relatively small subset group size, for instance 4 out of 24 animals, a KDE map can still be generated that is about 75% similar to that of the entire group, which may be useful in some cases. In fact, if the only parameter of interest is identification of heavily visited areas, it may be possible to use as few as four animals. If the goal of a study is to quantify cattle spatial occupancy and later correlate it with other data that have similar spatial resolutions as cattle movement data, such as soil characteristics and crop yields, it is recommended to use a larger subset group (e.g. 18 cows out of 24 cows) for monitoring in order to improve accuracy.

## Conclusions and Suggestions

Animal movement is a complex phenomenon that can be driven or affected by many factors such as animal internal states (e.g. body weight, perception and memory) and external environment (e.g. availability of forage and water, weather, predators and topography), which may be stochastic in nature. Application of GPS technology has provided useful data for tracking animal movement; however, previous studies may have not instrumented enough animals to provide accurate information of herd activities due to the cost of technology or other technical constraints (see [25] for a more comprehensive review of 99 cattle studies using GPS beginning in 1997 through 2012 as well as challenges in cost, accuracy, and data analysis). Appropriate subset group size for animal movement monitoring with GPS is dependent on specific research objectives. Our development of a computational approach for analyzing cattle movement data statistically evaluated the errors in subsequent location data analysis caused by monitoring subset groups of animals instead of the entire herd. Results showed monitoring an appropriate subset group of cattle could preserve most information with acceptable errors for subsequent analysis. On the one hand, it may be interesting to note that some parameters (i.e. average herd travel speed and daily travel distance) quantifying cattle movement were generally over predicted when subsampling from the herd, while the other parameter (i.e. average herd radius) was under predicted. On the other hand, heavily visited areas can be identified with very few data, while correlation to other environmental factors may require more data. We expect analogous results for similar experimental environments and herd sizes. Further, the computational approach we developed here can be applied to other systems (e.g. other livestock or wildlife) that utilize GPS units for animal movement monitoring, providing insights into future experimental designs when considering tradeoffs between data quality and costs. A limitation of this analysis results from the time consuming and great computing requirements in order to consider all possible combinations of subset groups regarding the group sizes. Based on this, a lesser number of randomly chosen instances were included for the analysis. Considering sufficient number of combinations of subset group sizes for this analysis remains a challenge for future work.

## Supporting Information

S1 File GPS data of Group A and B. (ZIP)

S2 File MATLAB codes and results. (ZIP)
